# Effects of alcohol and PARP inhibition on RNA ribosomal engagement in cortical excitatory neurons

**DOI:** 10.3389/fnmol.2023.1125160

**Published:** 2023-04-11

**Authors:** Harish R. Krishnan, Gian Paolo Vallerini, Hannah E. Gavin, Marina Guizzetti, Hooriyah S. Rizavi, David P. Gavin, Rajiv P. Sharma

**Affiliations:** ^1^Center for Alcohol Research in Epigenetics, Department of Psychiatry, University of Illinois at Chicago, Chicago, IL, United States; ^2^Department of Psychiatry, University of Illinois at Chicago, Chicago, IL, United States; ^3^Department of Behavioral Neuroscience, Oregon Health & Science University, Portland, OR, United States; ^4^VA Portland Health Care System, Portland, OR, United States; ^5^Jesse Brown Veterans Affairs Medical Center, Chicago, IL, United States

**Keywords:** TRAP-seq, ribosomal engagement, RNA sequencing, alcohol use disorder (AUD), PARP (poly(ADP-ribose) polymerase, ABT-888, insulin receptor signaling pathway

## Abstract

We report on the effects of ethanol (EtOH) and Poly (ADP-ribose) polymerase (PARP) inhibition on RNA ribosomal engagement, as a proxy for protein translation, in prefrontal cortical (PFC) pyramidal neurons. We hypothesized that EtOH induces a shift in RNA ribosomal-engagement (RE) in PFC pyramidal neurons, and that many of these changes can be reversed using a PARP inhibitor. We utilized the translating ribosome affinity purification (TRAP) technique to isolate cell type-specific RNA. Transgenic mice with EGFP-tagged Rpl10a ribosomal protein expressed only in CaMKIIα-expressing pyramidal cells were administered EtOH or normal saline (CTL) i.p. twice a day, for four consecutive days. On the fourth day, a sub-group of mice that received EtOH in the previous three days received a combination of EtOH and the PARP inhibitor ABT-888 (EtOH + ABT-888). PFC tissue was processed to isolate both, CaMKIIα pyramidal cell-type specific ribosomal-engaged RNA (TRAP-RNA), as well as genomically expressed total-RNA from whole tissue, which were submitted for RNA-seq. We observed EtOH effects on RE transcripts in pyramidal cells and furthermore treatment with a PARP inhibitor “reversed” these effects. The PARP inhibitor ABT-888 reversed 82% of the EtOH-induced changes in RE (TRAP-RNA), and similarly 83% in the total-RNA transcripts. We identified Insulin Receptor Signaling as highly enriched in the ethanol-regulated and PARP-reverted RE pool and validated five participating genes from this pathway. To our knowledge, this is the first description of the effects of EtOH on excitatory neuron RE transcripts from total-RNA and provides insights into PARP-mediated regulation of EtOH effects.

## 1. Introduction

Alcohol use disorder (AUD) affects about 10% of adults in the United States (SAMHSA, 2018). The prefrontal cortex (PFC) is a brain region thought to play an important role in alcohol seeking behavior (Stephens and Duka, 2008; Crews and Boettiger, 2009; Koob and Volkow, 2010; Willcocks and McNally, 2013; Gass et al., 2014; Brown et al., 2016; Cannady et al., 2017; Palombo et al., 2017). Prior studies have documented the important role of PFC excitatory pyramidal neurons in the reinforcing effects of alcohol (Willcocks and McNally, 2013; Gass et al., 2014; Brown et al., 2016; Cannady et al., 2017; Palombo et al., 2017). In rodents, the excitatory neurons in the prelimbic cortex mediate cue-mediated ethanol seeking behavior (Gass et al., 2014; Brown et al., 2016; Cannady et al., 2017). The CaMKIIα enzyme is characteristically expressed by cortical pyramidal neurons (Jones et al., 1994; Tighilet et al., 1998).

Poly-ADP Ribose Polymerase-1 (PARP1) is an enzyme that affects gene expression through histone modification and DNA methylation, among other molecular mechanisms (Kraus and Hottiger, 2013). In C57BL/6J male mice, we recently demonstrated that binge-like alcohol consumption induces PFC PARP mRNA expression and enzymatic activity (Vallerini et al., 2020). Furthermore, virally induced PARP1 overexpression in the PFC increased voluntary alcohol consumption, while the PARP inhibitor ABT-888 decreased it (Vallerini et al., 2020).

Our primary objective was to examine the effects of EtOH and of the co-administration of EtOH and ABT-888 on the distribution of RNA transcripts, specifically the subset that are captured by ribosomes for protein translation (ribosome-engaged, RE) from the total-RNA pool in excitatory PFC neurons. The addictive process is the result of a complex interplay among the various neural cell-types, including excitatory and inhibitory neurons, as well as glial cells. We hypothesized that EtOH induces a shift in RE in the PFC pyramidal neurons, and that many of these changes can be reversed using a PARP inhibitor.

The cell-type specificity of the study derives from the use of a transgenic mouse model that expresses a GFP-Rpl10a ribosomal protein under the control of the promoter for CaMKIIα, an enzyme expressed predominantly in excitatory pyramidal neurons (Jones et al., 1994). Ribosome-associated RNA (RE) from excitatory PFC pyramidal neurons was isolated by the translating ribosome affinity purification (TRAP) technique as previously reported (Drane et al., 2014). The RNA isolated using TRAP is a proximal step in protein expression since primarily transcripts targeted for translation will be isolated. There have been no studies, to date, that have investigated the effects of EtOH on the distribution of RE transcripts in a specific neural cell type.

## 2. Materials and methods

### 2.1. Transgenic mice

To obtain RNA for TRAP, we used a mouse strain in a C57BL/6J background that expresses a fluorescently tagged ribosomal protein (EGFP-Rpl10a) in the presence of tetracycline-controlled transactivator (tTA). TRAP mice for our study were derived by crossing C57BL/6J-Tg(tetO-EGFP/Rpl10a)5aReij/J with B6.Cg-Tg(CaMKIIα-tTA)1Mmay/DboJ(CaMKIIα-tTA) mice. Both mouse lines were purchased from Jackson Laboratories. The resulting offspring have ribosomes tagged with EGFP expressed only in CaMKIIα expressing cells (Heiman et al., 2008). Genotyping of mice was performed according to standard procedures.

### 2.2. Animal treatment and sample collection

All animal studies were conducted in accordance with the National Institutes of Health Guidelines for the Care and Use of Laboratory Animals. Mice were group housed on a standard 12 light/12 dark cycle and allowed *ad libitum* access to food and water. Male transgenic mice 8-12 weeks old were randomly assigned to three treatment groups: control, ethanol (EtOH), and ethanol + ABT-888 (EtOH + ABT-888). The animals were administered i.p. twice a day with normal saline (CTL) or ethanol (EtOH, to a final daily dose of 2 g/kg) for four consecutive days (2 h between the first and second injection of the day). The dosing strategy was the same as the involuntary ethanol administration protocol reported earlier (Vallerini et al., 2020). We simulated the drinking-in-the-dark binge drinking paradigm (this is a 4d drinking paradigm) in which, PARP inhibitor reduced drinking. The time point of ABT-888 administration was chosen since in our earlier study (Vallerini et al., 2020), we observed a change in drinking behavior after a single dose of ABT-888 was administered after a period of 4 days of EtOH administration. ABT-888 (25 mg/kg) was co-administered with ethanol on the fourth day in a sub-group of mice that received ethanol in the previous three days (EtOH + ABT). Two hours after the second injection on day 4, mice were sacrificed via rapid CO_2_ asphyxiation and decapitation (Supplementary Figure 4). Blood was rapidly collected from the trunk, centrifuged at 2500 RPM for 15 minutes to separate the serum, and kept at –80°C until analyzed; the brain was dissected on a brain block to isolate the PFC from the two hemispheres. This was based on coordinates from our earlier publication (Vallerini et al., 2020) (anterior/posterior: +1.9 mm; medial/lateral: ±0.5 mm; dorsal/ventral: –2.5 mm from Bregma). PFC was immediately homogenized and subject to immunoprecipitation-driven RNA extraction according to the TRAP protocol as well as total-RNA extraction for the INPUTs.

### 2.3. Blood ethanol concentration (BEC)

An enzymatic assay calibrated against a standard curve of ethanol known concentrations was utilized to determine BECs (Jung and Férard, 1978). Briefly, ethanol standard samples were prepared by mixing absolute ethanol and ultrapure water to yield a 1000 mg/dL ethanol stock solution. This solution was serially diluted to yield a set of eight standard samples in the range of 7.8–1,000 mg/dl ethanol. Perchloric acid (40 μl of 3.75%) was added to 10 μl of samples (serum) and standards and centrifuged for 6 minutes at 2000 RPM. The supernatant was retained. β-Nicotinamide adenine dinucleotide lithium salt from Saccharomyces cerevisiae (Sigma #N7132) was added at a final concentration of 2.5 mM, and Alcohol Dehydrogenase from Saccharomyces cerevisiae (Sigma #A7011) was added at a final concentration of 5 μM to samples and standards and incubated at 35°C for 40 minutes. BEC was calculated in milligrams per deciliter and noted as follows: control group (13.2 ± 18.9 mg/dl; *n* = 7), EtOH group (285 ± 84.2 mg/dl; *n* = 5) and EtOH + ABT-888 group (221 ± 113 mg/dl; *n* = 5).

### 2.4. Immunoprecipitation and RNA extraction (TRAP protocol)

The technology was developed in the Heintz lab at Rockefeller University (Heiman et al., 2008). Briefly, PFC samples were homogenized in the appropriate buffer (containing NP-40, KCl, Tris, MgCl2, cycloheximide, protease inhibitor cocktail, RNAse inhibitors and DTT) and centrifuged. Supernatant (40 μl) was collected for total-RNA (INPUT), and the remaining supernatant (approximately 400 μl) was transferred to a separate tube and treated with anti-EGFP antibodies and Agarose Plus A/G beads (Santa Cruz_sc-2003*) overnight. After centrifugation at 1200 RPM for 1 min, the supernatant was removed and the beads, containing ribosome-bound RNA (TRAP samples), were washed 4 times with a high-salt buffer solution (containing NP-40, KCl, Tris, MgCl2, cycloheximide, and DTT) and eluted with QIAZOL (Qiagen # 79306). TRAP-RNA and total-RNA samples were extracted using the miRNeasy Mini Kit (Qiagen # 217004), according to manufacturer’s instructions.

### 2.5. RNA-seq sample preparation and analysis

RNA samples were quantified using the Quantus fluorimeter (Promega) with dual RNA/DNA quantification. Levels of remaining DNA did not exceed 10% of the total amount of Nucleic Acid. RNA Integrity Numbers (RIN) were assessed using Agilent 4200 TapeStation (Agilent) (Mean:7.33; SD: 0.46; Min: 6.4; Max: 7.8). The samples were subjected to rRNA depletion and sequencing libraries prepared for Illumina sequencing (CORALL Total RNA-seq Library Prep Kit with Unique Dual Indices [Lexogen, PN M11696] with RiboCop HMR rRNA Depletion Kit V1.3 [Lexogen, PN K03796]). Final amplified libraries were purified, quantified and average fragment sizes confirmed to be 254 –323 and then subjected to test sequencing on a MiniSeq instrument in order to check sequencing efficiencies. Sequencing was carried out on NovaSeq 6000 (Illumina), SP flowcell, 2 × 50 bp PE reads.

The resulting FASTQ files were checked for adapters and following QC, raw reads were aligned to the reference genome, mm10, in a splice-aware manner using the STAR aligner (Dobin et al., 2013). The genome reference utilized ENSEMBL gene and transcript annotations, which included non-coding RNAs in addition to mRNAs. The expression level of the genes was quantified using FeatureCounts (Liao et al., 2014). Differential expression statistics (Log2 fold-change and p-value) were computed using edgeR (Robinson et al., 2010; McCarthy et al., 2012) on raw expression counts obtained from quantification. Prior to analysis, the counts were filtered to exclude any gene that either had less than a total of 500 counts across all samples or was detected in fewer than 3 samples. The data were normalized using the trimmed mean of m-values (TMM) normalization. The normalized data were modeled across treatment status, i.e., Control, EtOH or EtOH + ABT-888, as well as by sample type, i.e., Input or TRAP. The terms of the model were tested using the likelihood ratio test, i.e., glmLRT function, within edgeR. Pairwise tests of the expression data were computed using the exactTest function within edgeR. In all cases, adjusted p-values were computed using false discovery rate (FDR) correction (Benjamini and Hochberg, 1995). Significant genes were determined based on an FDR threshold of 0.01 or 0.05 in the comparison.

Six experimental samples were obtained from the three experimental conditions: Control_TRAP (*n* = 3); EtOH_TRAP (*n* = 4); EtOH + ABT-888_TRAP (*n* = 4); Control_total-RNA (*n* = 3); EtOH_total-RNA (*n* = 4); EtOH + ABT-888_total-RNA (*n* = 4). We further performed the following comparisons indexed in Table 1: (A1) Control_TRAP vs. Control_total-RNA; (A2) EtOH_TRAP vs. EtOH_total-RNA; (A3) EtOH + ABT-888_TRAP vs. EtOH + ABT-888_total-RNA; (B1) EtOH_TRAP vs. Control_TRAP; (B2) EtOH + ABT-888_TRAP vs. Control_TRAP; (C1) EtOH_total-RNA vs. Control_total-RNA; (C2) EtOH + ABT-888_total-RNA vs. Control_total-RNA. The first three comparisons in Table 1 (A1, A2, and A3) utilized total-RNA measurements for scaling Ribosome engagement (RE), similar to the published RE method (Ouwenga et al., 2017; Rodrigues et al., 2020). We also performed comparisons entirely within the cortical samples used for both TRAP RNA (B1 and B2) and total-RNA (C1 and C2). Gene lists were generated from these comparisons using an FDR < 0.01 (A1, A2, A3) or 0.05 (B1, B2; C1, C2) and subjected to an overrepresentation analysis for biological pathways using Ingenuity Pathway Analysis (IPA^®^; QIAGEN).

**TABLE 1:** Summary of differentially expressed transcripts (DETs) using Log2 fold changes at FDR < 0.05, and parsed from 14,073 genes based on seven comparisons in the PFC.

Comparisons	DET (FDR<0.05)	UP	DOWN
**(A) Distribution of transcripts: Ribosomal and Total pool**			
(A1) Control_TRAP/Control_INPUT	8,698	4,279	4,419
(A2) EtOH_TRAP/EtOH_INPUT	7,421	3,573	3,848
(A3) (EtOH + ABT-888)_TRAP/(EtOH + ABT-888)_INPUT	6,200	2,923	3,277
**(B) Ribosomal transcripts: Treatments normalized to control**			
(B1) EtOH_TRAP/Control_TRAP	1,365	718	647
(B2) (EtOH + ABT-888)_TRAP/Control_TRAP	316	142	174
**(C) Total RNA expression by treatment**			
(C1) EtOH_INPUT/Control_INPUT	3,015	1,517	1,498
(C2) (EtOH + ABT-888)_INPUT/Control_INPUT	544	343	201

“UP” and “DOWN” indicate number of transcripts that are higher or lower in the numerator sample of the indexed ratio noted in column one.

IPA Pathway lists were prioritized by computing −log *p*-values and Z scores to identify known biological pathways that appeared most significantly affected by the genes in the data set. The significance values (−log *p*-value of overlap) for the canonical pathways indicate the probability of association of molecules from our dataset with the canonical pathway by random chance alone. IPA assigns the negative −log *p*-value to pathways based on a Fisher’s exact test of the probability of the number of molecules from the user-created dataset included in the given pathways vs. being included based on chance alone. Z-scores measure the inclusion/exclusion of genes within networks according to experimental conditions and indicate the activation/inhibition states of the molecule. Gene lists were uploaded to IPA with Ensemble ID, Log2 FC and FDR values and the resulting Canonical Pathway information along with –log p-values obtained.

### 2.6. Quantitative real-time reverse transcription polymerase chain reaction and statistics

The RNA used for sequencing was utilized for cDNA preparation and target gene validations by qRT-PCR using PikoReal Real-Time PCR (Thermo Fisher, Waltham, MA, USA) and Maxima^®^ SYBR Green/ROX qPCR Master Mix (Thermo Fisher, Waltham, MA, USA). To confirm amplification specificity, the PCR products were subject to a melting curve analysis, in which only one peak was observed. Crossing point values were measured with the PikoReal analysis software. Primers (Supplementary Table 1) were designed to span at least one intron exon boundary. The following cycling conditions were used: 10 min at 95°C then 40 cycles at 95°C for 30 s, 60°C for 1 min, and 72°C for 30 s. For normalization of mRNA expression, the geometric mean of Gapdh and β-Actin was used. Relative fold changes were determined using the ΔΔCt method.

Statistical significance was determined using one-way analysis of variance (ANOVA) followed by *post-hoc* Bonferroni or Tukey tests or with Student’s *t*-test as appropriate. All statistical analyses were performed using GraphPad Prism for Windows (GraphPad Software, San Diego, CA, USA)^1^ or SPSS (IBM Corp. released 2016, IBM SPSS Statistics for Windows, version 24.0, Armonk, NY, USA).

### 2.7. Validation of translating RNA from CaMKIIα-expressing cell isolation using gene-specific expression

In preliminary TRAP experiments we measured several markers that would confirm the successful isolation of RNA from CaMKIIα-expressing cells. Specifically, we measured *Egfp*, *CamKII*α, *S100b*, and *Ef101557* expression in TRAP samples. We expected isolated ribosomes to have high green-fluorescent protein mRNA expression (*Egfp*) and *CamKII*α expression and low expression of the astrocyte specific-*S100b* and microglial-specific *Ef101557* transcripts. In support of the ability of the TRAP technique to isolate *CaMKII*α-expressing cells we found *Egfp* (2.654 vs. 0.790, t_6_ = 2.547, *p* = 0.04, *n* = 4 per group) and *CamKII*α (1.416 vs. 0.265, t_4_ = 6.022, *p* = 0.004, *n* = 3 per group) to be highly elevated in the TRAP samples compared to total-RNA, while astrocyte marker *S100b* (2.624 vs. 1.715, t_6_ = 3.357, *p* = 0.02, *n* = 4 per group) and microglial marker *Ef101557* (29.985 vs. 25.121, t_6_ = 6.063, p = 0.0009, n = 4 per group) were reduced.

## 3. Results

### 3.1. Effects of EtOH and ABT-888 on RE and total-RNA in PFC neurons

#### 3.1.1. RE/total-RNA ratio

**FIGURE 1:**
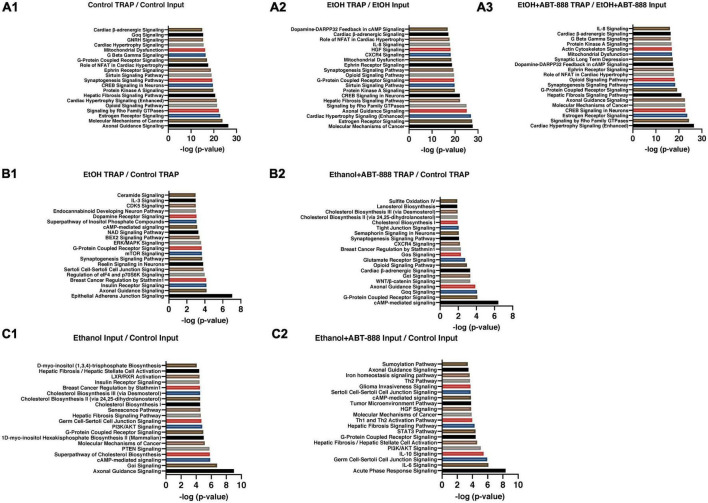
Top IPA canonical pathways enriched in gene lists from Table 1. The top 20 significantly enriched canonical pathways based on IPA coordinated with Table 1. **(A)** TRAP-seq (RE fraction) compared to RNA-seq (total-RNA) for control, EtOH treated, and ABT-888 treated. Panels **(A1–A3)** from Table 1. **(B)** TRAP-seq of EtOH treated compared to control, and ABT-888 treated compared to control. Panels **(B1, B2)** from Table 1. **(C)** RNA-seq of EtOH treated compared to control and ABT-888 treated compared to control. Panels **(C1, C2)** from Table 1. The pathways are ranked according to IPA generated –log *p*-value scores, and the pairwise comparisons were sorted using FDR < 0.01 for panels **(A1–A3)** comparisons and FDR < 0.05 for panels **(B1–C2)** comparisons.

In this study, total-RNA is not cell-type specific (multiple cell-types), as is TRAP-RNA, but can still provide valuable context of overall transcriptional activity in this PFC region. Because, both total-RNA and TRAP-RNA are extracted from the same sample, we can contextualize the background from which the ribosome captures RNA. We examined the distribution of RNA transcripts between the ribosomal fraction in CaMKIIα-positive excitatory neurons (TRAP samples, RE) and total-RNA. The first three rows of Table 1 (lines A1, A2, and A3) present the number of transcripts that are undergoing RE compared to total-RNA for each condition (visualized as volcano plots in Supplementary Figure 1). In Table 1-A1, we note that in the Control condition (or absence of EtOH), out of 8698 transcripts, 4279 were significantly more likely to be engaged with ribosomes. A similar assessment in the independent EtOH and EtOH + ABT-888 samples (Table 1-lines A2 and A3, respectively) indicate an approximately equivalent percentage of transcripts that are significantly more engaged with ribosomes when compared to total-RNA; in the EtOH treatment (3573/7421) and EtOH + ABT-888 (2923/6200). We analyzed the “genome-wide” ratio of EtOH/EtOH + ABT-888 and could not recover FDR at the < 0.05 level. We considered the reasons, including using EtOH as the comparator renders a vigorously modified baseline upon which the addition of ABT-888 no longer provides “genome-wide” significance as can be seen from the significant perturbations induced by EtOH itself (up or down) in Table 1 (Lines A2 and A3). Nevertheless, we used a less stringent threshold (unadjusted *p* < 0.05) and performed pathway analyses and have included that information in Supplementary Table 2. This preliminary analysis also suggests that the percentage of RE transcripts are approximately similar between EtOH and EtOH + ABT-888, suggesting no systematic EtOH induced bias towards or against RE. The top canonical pathways emerging from each of the comparisons (A1, A2, and A3) are shown in Figures 1A1–A3.

#### 3.1.2. RE ratio

RE is dependent upon neural cell-type, the rate of genomic expression, mRNA stability, the presence of ribosome-binding motifs and their electrostatic properties, and cellular location (nucleus, cytoplasm, synaptosomes). Given these considerations, we next restricted our analysis to only TRAP samples. Administration of EtOH caused the differential distribution of 1,365 ribosomal transcripts (EtOH_TRAP vs Control_TRAP; FDR < 0.05) across the pool of RE transcripts; 718 transcripts had a higher probability of RE, and 647 had lower RE (Table 1-B1; shown as a volcano plot in Supplementary Figure 1). Enriched canonical pathways emerging from this list included “Epithelial Adherens Junction Signaling,” “Axonal Guidance Signaling,” and “Insulin Receptor Signaling” (Figure 1B1). In a subset of TRAP mice that received EtOH we co-administered the PARP inhibitor ABT-888 (EtOH + ABT-888). This resulted in a much lower number of 316 transcripts differentially altered compared to control (EtOH + ABT-888_TRAP vs Control_TRAP; FDR < 0.05; Table 1-B2) out of which, 174 were downregulated and 142 upregulated (shown as a volcano plot in Supplementary Figure 1). The top enriched IPA pathways in this list of 316 genes, were “cAMP mediated Signaling,” “G-Protein Coupled Receptor Signaling,” and “Gαq Signaling” (Figure 1B2). Interestingly, of these 316 transcripts, 250 were also significantly altered by EtOH alone (in the same direction of change in every case), while 66 were novelly altered by the EtOH + ABT-888 condition. Hence, we get 1115 transcripts (1365-250), or 82% (1115/1365) of all EtOH affected genes whose ethanol-induced expression level can be said to be “reversed” or “negated” by ABT-888 co-administration. We elaborate on this in section “3.4. Reversal by ABT-888 on RE transcripts altered by EtOH” (below).

#### 3.1.3. Total RNA ratio

Following a similar analysis method as described in section “3.1.2. RE ratio,” we examined the EtOH total-RNA samples compared to Control total-RNA samples. EtOH induced 3,015 genes to be differentially expressed compared to control (1517 higher and 1498 lower) (EtOH_INPUT vs Control_INPUT; FDR < 0.05; Table 1-C1; shown as a volcano plot in Supplementary Figure 1). In this gene list the top enriched IPA pathways were “Axonal guidance Signaling,” “Gαi Signaling,” and “cAMP-mediated Signaling” (Figure 1C1). The addition of ABT-888 (EtOH + ABT-888) manifested a redistribution of far fewer transcripts than were altered by EtOH alone compared to control. There were 544 transcripts (343 upregulated and 201 downregulated) (EtOH + ABT-888_INPUT vs Control_INPUT; FDR < 0.05; Table 1-C2; shown as a volcano plot in Supplementary Figure 1). Enriched pathways include “Acute Phase Response Signaling,” “IL-6 Signaling,” and “Germ Cell-Sertoli Cell Junction Signaling” (Figure 1C2). Interestingly, 501 of the 544 ribosome-bound transcripts that were altered by EtOH + ABT-888 were also altered by EtOH alone and in every case, in the same direction of change. Hence, we get 2514 transcripts (3015-501), or 83% (2514/3015) of all EtOH affected genes whose ethanol-induced expression level can be said to be “reversed” or “negated” by ABT-888 co-administration. Moreover, EtOH + ABT-888 altered 43 transcripts that were not affected by EtOH alone suggesting novel regulatory mechanisms. Overall, the data indicate that addition of ABT-888 is predominantly able to “reverse” (83%) the effects of EtOH on total-RNA.

### 3.2. EtOH and ABT-888 modulation of RE and total-RNA enriched pathways

**FIGURE 2:**
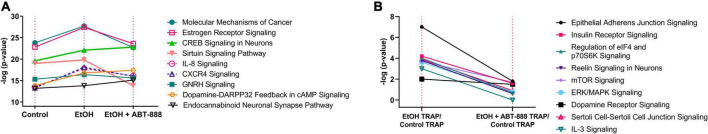
Modulation of enriched signaling pathways between experimental conditions. The –log p-value scores (obtained from IPA) of nine selected pathways are plotted, showing their activation patterns between the experimental conditions in Table 1. **(A)** RE (TRAP-seq) compared to total-RNA (RNA-seq) in Control, EtOH treated, and EtOH + ABT-888 treated (lines A1, A2, and A3 from Table 1). **(B)** Comparing RE only of EtOH treated and EtOH + ABT-888 treated (lines B1 and B2 from Table 1).

With the initial hypothesis that EtOH will induce a shift in RE transcripts which ABT-888 may reverse, we proceeded to identify shifts in IPA pathways between the experimental conditions. From Figure 1, we identified the top 20 canonical pathways based on IPA generated −log *p*-value scores in each computed ratio, and further identified a subset of pathways that were common (Figure 2) across the indexed conditions (A1 vs. A2 vs. A3; and B1 vs. B2). In Figure 2A where pathways are scaled to total-RNA, we note “Molecular Mechanisms of Cancer” and “Estrogen Receptor Signaling” pathways show a strong induction with EtOH following a more suppressive/normalization state with ABT-888. Likewise, IL-8 Signaling and CXCR4 Signaling show a strong induction which appears to reverse with ABT-888. We next examined modulation of these pathways in only TRAP samples across the experimental conditions by comparing lines B1 and B2 from Table 1 and are presented in Figure 1B, of which “Epithelial Adherens Junction Signaling,” “Insulin Receptor Signaling,” and “Regulation of elF4 and p70S6K Signaling,” are the top 3 (Figure 2B). Comparing the two conditions (B1 and B2), we observed that most of the top 20 enriched pathways had an identifiable downward shift in RE after ABT-888 treatment (Figure 2B).

### 3.3. Evaluating coordinated changes between the RE and total-RNA pool

**FIGURE 3:**
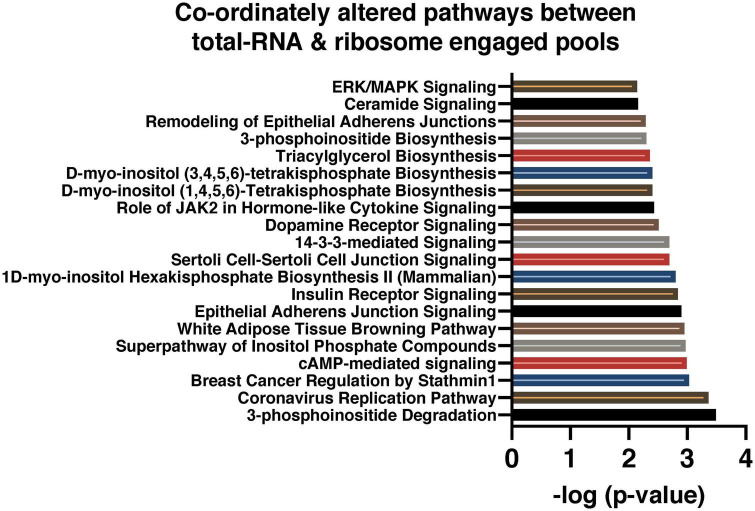
Signaling pathways predicted by IPA in coordinately altered transcripts between RE and total-RNA pool. These are the top twenty canonical pathways from among the differentially expressed transcripts that were common to both RE (from TRAP-seq data) and total-RNA (from RNA-seq data), at an FDR < 0.05. The pathways are ranked according to IPA generated –log p-value scores.

We analyzed the data set from TRAP (translating) and total-RNA (transcribed) to detect RNA transcripts that are significantly enriched or reduced in both ribosomes and the total-RNA pool. We identified 785 transcripts significantly altered (FDR < 0.05) in both the RE and total-RNA pools, suggesting coordinated changes in both transcription and translation regardless of cell type. These changes were in the same direction in all but three instances. Expression of Fbln5 was increased with EtOH in the total-RNA pool and decreased in the RE pool, while expression of Pltp6 and Ptpn6 was decreased with EtOH in the total-RNA pool and increased in the RE pool. We next used IPA to identify enriched canonical pathways in this data set of 785 transcripts. The top 20 pathways ranked according to their negative log p-value are shown in Figure 3 and included “3-Phosphoinositide Degradation,” “Coronavirus Replication Pathway,” and “Breast Cancer Regulation by Stathmin 1.”

### 3.4. Reversal by ABT-888 on RE transcripts altered by EtOH

**FIGURE 4:**
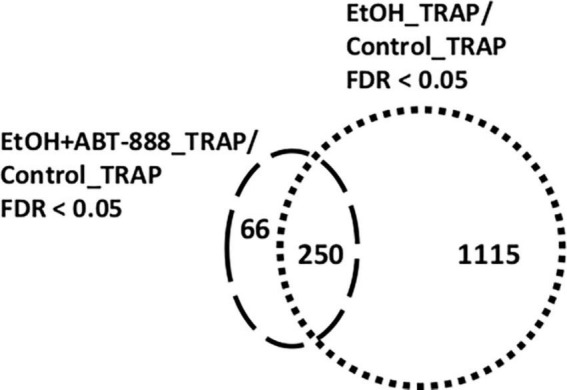
Venn diagram illustrating the differentially expressed transcripts identified in the different datasets. In RE (TRAP-seq) samples, administration of EtOH caused the differential distribution of 1,365 ribosomal transcripts (Table 1; B1) (EtOH_TRAP vs Control_TRAP; FDR < 0.05) represented by the “dotted” circle. Co-administering the PARP inhibitor ABT-888 (EtOH + ABT-888) resulted in 316 transcripts differentially altered compared to control (EtOH + ABT-888_TRAP vs Control_TRAP; FDR < 0.05; Table 1-B2) represented by the “dashed” circle. Out of these 316 transcripts, 66 were uniquely altered by EtOH + ABT-888, but not EtOH, whereas 250 transcripts were altered by both EtOH and EtOH + ABT-888. This yielded 1,115 transcripts (1365-250) that were altered exclusively by EtOH and no longer by ABT-888 (or “reversed by ABT-888”) compared to Control.

**FIGURE 5:**
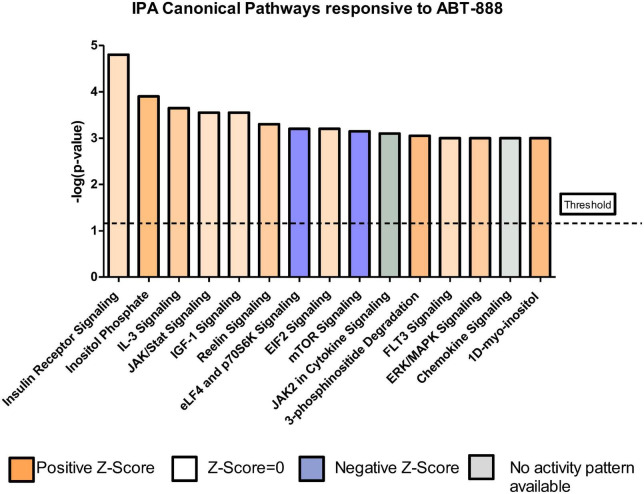
Top canonical pathways emerging from the data where the effects of ethanol are reversed with ABT-888 in RE samples. IPA identified 15 significant canonical pathways (ranked based on –log *p*-value) in the list of 1115 transcripts from the RE (TRAP-seq) data that were ‘reversed by co-administration of EtOH + ABT-888’. Blue = negative z-score = –2 and impact on the pathway; orange = positive z-score = +2 and impact on the pathway; white = z-score = 0, signifying no impact on the pathway.

We wanted to identify the number of transcripts whose EtOH-induced expression changes were nullified or “reversed” by the addition of ABT-888. We identified 1115 (1365-250) transcripts or 82% (1115/1365) of all EtOH affected genes to be “reversed” by ABT-888 co-administration (shown as a volcano plot; Supplementary Figures 2, 3). A schematic representation is shown in Figure 4. These genes were analyzed by IPA to identify enriched pathways and the 15 highest-ranking canonical pathways are indicated in Table 2 and Figure 5. We observed “Insulin Receptor Signaling” emerging as the top ranked canonical pathway (Table 2), suggesting that ethanol’s effects on this pathway are regulated by PARP signaling.

**TABLE 2:** Top 15 canonical pathways predicted by IPA emerging from the 1,115 transcripts that are reversed with ABT-888.

Top 15 canonical pathways	Number of genes normalized by ABT-888	List of genes normalized by ABT-888
Insulin receptor signaling	19	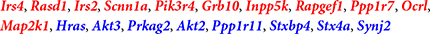
Superpathway of inositol phosphate compounds	22	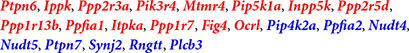
IL-3 signaling	12	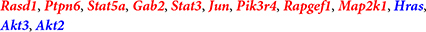
JAK/Stat signaling	12	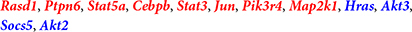
IGF-1 signaling	13	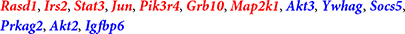
Reelin signaling in neurons	15	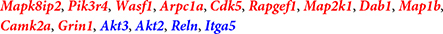
Regulation of eIF4 and p70S6K signaling	18	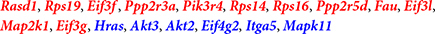
EIF2 signaling	22	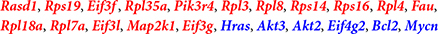
mTOR signaling	21	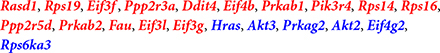
Role of JAK2 in hormone-like ytokine signaling	7	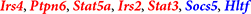
3-Phosphoinositide degradation	17	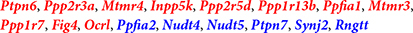
FLT3 signaling in hematopoietic progenitor cells	11	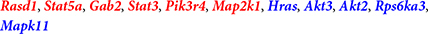
ERK/MAPK signaling	20	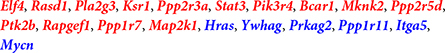
Chemokine signaling	11	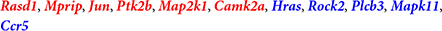
1D-Myo-inositol hexakisphosphate biosynthesis II (mammalian)	5	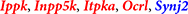

Transcripts that were significantly altered by EtOH, but not by co-administration of EtOH plus ABT-888. The genes indicated in red are up-regulated and genes indicated in blue are down-regulated by EtOH compared to control.

### 3.5. Candidates for validation from insulin receptor signaling and chemokine signaling

**FIGURE 6:**
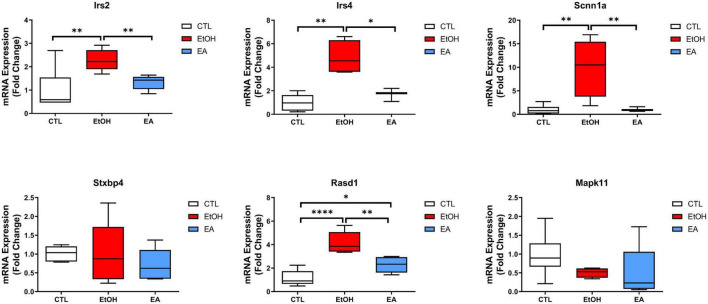
Validation of TRAP-seq results on selected genes. qRT-PCR analysis of six genes selected from Insulin Receptor Signaling pathway (Irs2, Irs4, Scnn1a, Stxbp4, and Rasd1) and Chemokine Signaling pathway (Mapk11). Relative expression (Fold Change) is compared between TRAP_EtOH/TRAP_CTL and TRAP_EtOH + ABT-888/TRAP_CTL, respectively, using the mean fold-change for Irs2 (FC = 2.28; *p* = 0.0093, FC = 1.33; *p* = 0.43), Irs4 (FC = 4.82; *p* = 0.0011, FC = 1.70; *p* = 0.19), Scnn1a (FC = 9.86; *p* = 0.0075, FC = 0.95; p = 0.95), Stxbp4 (FC = 1.04; *p* = 0.96, FC = 0.72; *p* = 0.15), Rasd1 (FC = 4.17; *p* < 0.0001, FC = 2.29; *p* = 0.02), and Mapk11 (FC = 0.51; *p* = 0.15, FC = 0.50; *p* = 0.25). Results are represented as the mean fold-change normalized to the geometric mean of the housekeeping genes and error bars indicate the range of relative expression calculated using 2^– (Δ^ ^Δ^ ^Ct ± standard^ ^deviation)^. *p < 0.05, ^**^p < 0.01, ^****^p < 0.0001.

We elected to validate six genes present in the 15 highest-ranking enriched canonical pathways emerging from the ethanol-regulated and PARP-reverted RE pool (Table 2 and Figure 5). The genes *Irs2*, *Irs4*, *Scnn1a*, *Stxbp4*, *Rasd1* (Insulin Receptor Signaling), and *Mapk11* (Chemokine Signaling) were chosen based on the differences in logFC between the EtOH_TRAP/Control_TRAP and the EtOH + ABT-888_TRAP/Control_TRAP comparisons. Genes showing a ΔlogFC value higher than 0.50 were selected for qRT-PCR validations (Table 3). Validation results confirmed our hypothesis of ABT-888 induced reversal of EtOH dysregulation of *Irs2*, *Irs4*, *Rasd1*, and *Scnn1a* (Figure 6). In the case of *Rasd1*, although ABT-888 was able to reverse the effects of EtOH, it was not reduced to control levels. All these validated targets, except Mapk11, lie within the Insulin Receptor Signaling pathway as noted in Table 2.

**TABLE 3:** Genes with the highest magnitude of reversal with coadministration of EtOH and ABT-888, corresponding to a difference in logFC > 0.50.

Gene name	logFC (TRAP-EtOH/TRAP-CTL)	logFC (TRAP-EtOH + ABT/TRAP-CTL)	Δ logFC
*Ccr5*	−4.52	0.02	4.55
*Irs4*	2.69	0.47	2.21
*Ptpn6*	2.07	0.91	1.16
*Mycn*	−2.24	−1.35	0.89
*Hltf*	−1.4	−0.55	0.84
*Pla2g3*	1.9	1.06	0.84
*Mapk11*	−2.28	−1.48	0.8
*Rasd1*	2.1	1.5	0.61
*Irs2*	1.28	0.7	0.59
*Scnn1a*	1.27	0.71	0.56
*Rngtt*	−1.24	−0.69	0.55
*Stxbp4*	−0.85	−0.3	0.55
*Mapk8ip2*	1.05	0.51	0.54

ΔlogFC: difference between the logFC in the TRAP_EtOH compared to TRAP_CTL and the TRAP_EtOH + ABT-888 compared to TRAP_CTL.

## 4. Discussion

We utilized TRAP-seq to measure genome-wide changes in the ribosome-bound RNA population (translatome) within excitatory pyramidal cells from the PFC in mice exposed to EtOH. Using the TRAP method, we isolated ribosome bound RNA from these cells. We demonstrated, firstly, that this fraction of RE transcripts (extracted from pyramidal cells) is enriched for CAMKIIα expression. Further, we demonstrated the impact of EtOH exposure on the transcript distribution in both the RE and total-RNA pools. Finally, in a direct test of the PARP hypothesis from our lab (Vallerini et al., 2020), we demonstrated that a PARP antagonist (ABT-888) can reverse the effects of EtOH. While this reversal is evident in both fractions, i.e., RE and total-RNA pools, we focused on the normalized RE transcripts to avoid unparameterized variations in the total-RNA pool (elaborated below).

Previous studies indicate that ABT-888 reduces consumption in the binge-like drinking-in-the-dark (DID) binge-like alcohol drinking model and PARP1 overexpression in the PFC increases DID alcohol drinking behavior (Vallerini et al., 2020). We hypothesized that EtOH will induce a shift in translating RNA in the PFC pyramidal neurons, and that many of these changes can be reversed using a PARP inhibitor. The most striking outcome of our study is that the addition of PARP inhibitor ABT-888 reversed 82% of the EtOH-induced changes in transcript levels in the RE samples and 83% of changes in the total-RNA samples with few novel transcripts being affected by the addition of ABT-888 (43 transcripts in total-RNA and 65 in RE samples).

Pathway analyses by IPA showed an overrepresentation of many interesting pathways and we utilized IPA generated –log p-value scores to visually compare the representation of these pathways across treatment conditions (Figure 2). Many interesting pathways emerged, most notably “CREB Signaling in Neurons,” which showed an increased IPA generated –log p-value score with EtOH administration. CREB mediated signaling has been shown to alter neuronal plasticity via epigenomic regulatory mechanisms in response to alcohol and other drugs of abuse resulting in a range of drug-induced behavioral phenotypes (Pandey et al., 2003; Levine et al., 2005; Wang et al., 2009; Krishnan et al., 2014). Interestingly, treatment with ABT-888 didn’t change the IPA generated −log *p*-value score of this network (“CREB Signaling in Neurons”). Given that PARP proteins are involved in DNA repair and chromatin remodeling, increased CREB signaling after administration of ABT could provide an alternate mechanism for a controlled and targeted shift in the translatome. Another interesting pathway was “Sirtuin Signaling Pathway,” which showed a decreased IPA generated –log p-value score, following administration of ABT-888. Sirtuins are described as NAD (nicotinamide adenine dinucleotide)-dependent protein deacetylases with myriad metabolic roles and interact with PARP to regulate concentrations of cellular NAD. PARP enzymes can be activated (acetylated) by p300/CBP and deactivated (deacetylated) by SIRT1. Our observation of a suppressed sirtuin pathway following ABT-888 demonstrates the intricate connection of the sirtuin family of enzymes and PARP activity. Thus, members of the Sirtuin and CREB signaling pathways could be exerting a shift in the translatome through nucleosomal DNA in a NAD-dependent manner.

**FIGURE 7:**
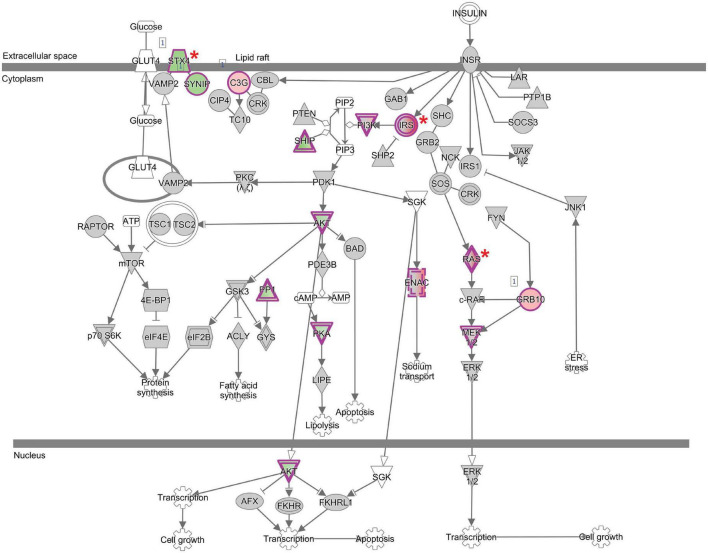
Functional network analysis of the Insulin Receptor Signaling Pathways generated by IPA. Gene names are displayed by cellular localization (extracellular space, cytoplasm, or nucleus). Interactions between molecules are supported by information in the Ingenuity knowledge base. Genes shaded red are upregulated and genes shaded green are downregulated. Genes marked with asterisk have been validated. Solid line ending with (a) solid arrowhead indicates activation; (b) vertical line indicates inhibition; (c) solid arrowhead plus vertical line indicates inhibits and acts on; (d) open arrowhead indicates translocation; (e) open diamond head indicates enzyme catalysis. Node shapes represent functional classes: concentric circles; complex/group, vertically diamonds; enzyme, horizontally ellipses; transcriptional regulator, triangle; phosphatase, inverted triangles; kinase, hexagon; translational regulator, trapezoid; transporter, inverted trapezoid for miRNA, and circles; other.

We focused on the Insulin Receptor Pathway (Figure 7) given that it was the strongest signal in the IPA analysis in the transcripts “normalized” by the PARP inhibitor ABT-888. Insulin is transported into the central nervous system through the blood brain barrier via a saturable mechanism and ligands to the insulin membrane receptor present in a variety of CNS cells. Insulin receptors are enriched in neurons, particularly in the synapse (Chiu and Cline, 2010), and are instrumental in synapse architecture. In this context, insulin ligand binding can recruit both excitatory (NMDA) and inhibitory receptors (GABA) (Chiu and Cline, 2010; Korol et al., 2018). Insulin Receptor Substrate 2/4 (Irs2 and Irs4) mediate the effects of insulin and insulin-like growth factor, are activated by tyrosine and serine kinases (Lee and White, 2004; Sadagurski et al., 2014) and trigger a kinase cascade including the downstream activation of AKT2/3 (Taguchi et al., 2007), noted in our results and presented as Supplementary Figure 3. Downstream, the signal bifurcates along kinase streams, including the MAP kinase and PI3K pathways. Our results suggest that ABT-888 may regulate molecules in the MAP kinase stream (Mapk11, Mapk8ip2; Tables 2, 3). Interestingly among the top 15 pathways from the ABT-888 normalized transcripts there are already well documented interactions. Insulin signaling can activate the mTOR pathway through the activation of PI3K/Akt signaling. mTOR also regulates insulin signaling by modulating the activity of insulin receptor substrate-1 (IRS-1) (Nakamura et al., 2022). Similarly, IGF-I can activate the mTOR pathway through the activation of PI3K/Akt and Ras/MAPK signaling (Mendoza et al., 2011). EtOH inhibits insulin receptor signaling both in the brain and liver at multiple levels (de la Monte et al., 2012). Chronic alcohol exposure has been shown to activate neuronal JAK/Stat signaling and also IGF-1 signaling (Rachdaoui and Sarkar, 2017). Our pharmacological intervention was to antagonize the effects of the PARP enzymes upon EtOH administration. PARP enzymatic activity is triggered by DNA damage, which is a common consequence of a host of reactive endogenous metabolites and the spontaneous decay of DNA. PARP1 repair/activity will induce a reorganization of local chromatin structure, especially in its ability to “parylate” histone proteins, nucleosome disassembly and chromatin relaxation. Consequently, EtOH effects via PARP activity on chromatin and other proteins can be considered to impact both transcription (via chromatin structure) and post-translational modification of non-histone proteins (via “parylation” of lysine residues) (Vallerini et al., 2020).

Poly (ADP-ribose) polymerase enzymes target numerous epigenetic proteins (HDAC5, HDAC7, TP2, G9a, and HSPA2, HP1) as well as transcription factors (PPARg, TIF1, TTF1, YY1, Smad) (Ali et al., 2016). One path of convergence between alcohol, insulin sensitivity, and PARP inhibition is noted by the efficacy of PPARg agonists to reverse this alcohol-induced insulin resistance, and this recalls the effects of the PARP inhibitor ABT-888, which executes the epigenetic modifications on PPARg promoters amongst other genes (de la Monte et al., 2012).

Although we could not profile coordinate changes in protein manufacture, TRAP-seq correlates highly with fold changes in large protein datasets in cell studies (∼2,500 protein using liquid chromatography/tandem mass spectrometry) (Rodrigues et al., 2020). Furthermore, because TRAP-seq appears to preferentially reveal the enrichment of genes with expression restricted to the isolated cell type, it may be less sensitive to genes with a broader expression pattern, i.e., even in non-pyramidal cells (Allison et al., 2015). Given the complexity of the novel TRAP paradigm, these initial experiments were designed to avoid behavioral or contingent variability and sought to establish a primary pharmacological effect of ethanol and ABT-888 on ribosome attachment. The objective was to establish pharmacological parameters prior to conducting contingent or behavioral experiments. We have previously shown in Vallerini et al., 2020 that both a voluntary (Drinking in the Dark) and an involuntary (i.p. administration) did not manifest in differences in BEC levels. A specific limitation in our study is the lack of a reference control (TRAP depleted RNA) to verify the depletion of the pyramidal transcriptome in this sample, which can then serve as reference data for the differential analysis. We attempted to address this limitation by evaluating coordinate changes in the RE and total-RNA pools (Figure 3). Additionally, evaluation of cellular function was not performed in the present study which would require generating TRAP models at the cellular level, a challenge we anticipate for the future. Furthermore, given the small number of genetically modified TRAP cells we were not able to design protein experiments given the tissue requirements. Alternate approaches such as genetic models to target protein pathways could be considered in separate experiments. Our future research will focus on motifs within RNA transcripts that engender ribosomal capture. Furthermore, in this study we used only male rodents and in future studies we plan to incorporate female rodents, which will allow us to merge the data and look for both shared and sex-specific changes. Genomic RNA expression is a molecular event using covalent modifications for nucleotide synthesis and is highly regulated at the promoter level. However, ribosome attachment is similar to ligand-receptor binding, non-covalent in nature, and likely propelled by biophysical considerations such as the minimization of free energy. From the transcriptional domain to the translational domain, this mapping is an exciting new area of research. We speculate that EtOH, with its wide cellular response, is an ideal experimental platform.

This study found that the addition of ABT-888 reversed a significant portion of the changes in transcript levels induced by ethanol, suggesting that PARP inhibitors may be able to target the molecular mechanisms underlying the development of alcohol addiction. We and others have previously reported on the role of PARP enzymes in drug-induced phenotypes. Both Cocaine and Ethanol significantly increase PARP1 expression and enzymatic activity in the nucleus accumbens and PFC respectively, and regulation of PARP inhibition modifies both cocaine and alcohol related behaviors (Scobie et al., 2014; Vallerini et al., 2020). Of particular relevance to the findings of this study is that PARP inhibition with ABT-888 reverses both the observed biomolecular effects of alcohol as well as reduces alcohol drinking (Vallerini et al., 2020). Considering that PARP inhibitors are clinically used in cancer (Dias et al., 2021), our findings would support the use of PARP inhibitors in alcohol use disorder.

## Data availability statement

The data presented in this study are deposited in the GEO repository, accession number GSE227947.

## Ethics statement

The animal study was reviewed and approved by VA Innovation and Research Review System (VAIRRS) Subcommittee on Research Safety and Security (SRSS) Animal Component of Research Protocol (ACORP).

## Author contributions

HK contributed to the data analysis, bioinformatics interpretation, pathway analysis, and manuscript preparation. GV conducted the experimental procedures and the early phase of data analysis. HG contributed to the editing, verification, and formatting. MG contributed to conceptualization and feasibility analysis. HR contributed to the pathway analysis, bioinformatics, and manuscript preparation. DG was responsible for the conceptualization, funding, and contributed toward data analysis and manuscript preparation. RS coordinated the interpretation, data analysis, and manuscript preparation and submission. All authors contributed to the article and approved the submitted version.

## Footnotes

1
www.graphpad.com
