# Supplementary material for: Effects of alcohol and PARP inhibition on RNA ribosomal engagement in cortical excitatory neurons
Source: Front Mol Neurosci. 2023 Apr 11;16:1125160. doi: 10.3389/fnmol.2023.1125160 (PMC10126255; doi:10.3389/fnmol.2023.1125160)
Supplement: Supplementary file 1 [file Data_Sheet_1.docx]

**Supplementary Materials**

**Effects of alcohol and PARP inhibition on RNA ribosomal engagement in cortical excitatory neurons**

Harish R. Krishnan, Gian Paolo Vallerini, Hannah E. Gavin, Marina Guizzetti, Hooriyah S. Rizavi, David P. Gavin & Rajiv P. Sharma

**Supplementary Results:**

Control_TRAP/Control_INPUT EtOH_TRAP/EtOH_INPUT


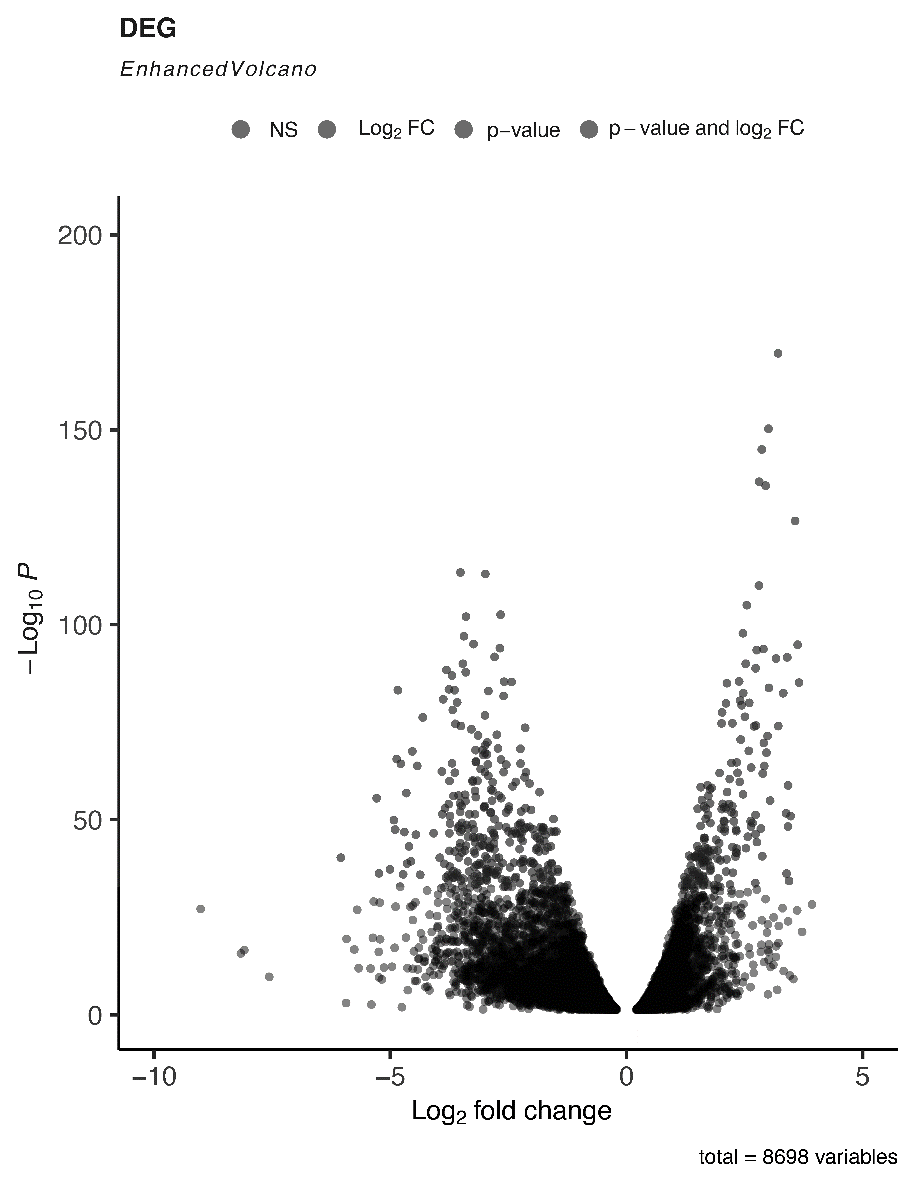

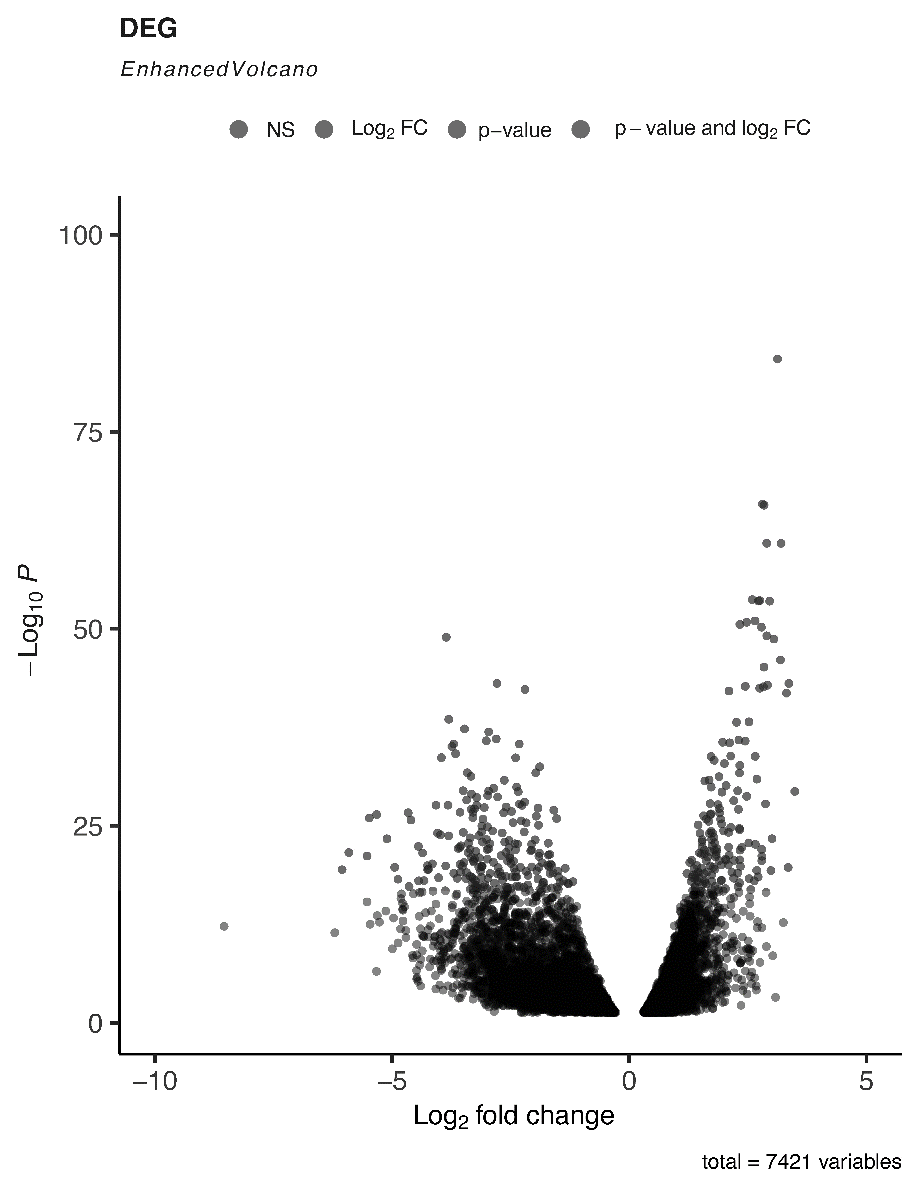


(EtOH+ABT-888)_TRAP/(EtOH+ABT-888)_INPUT


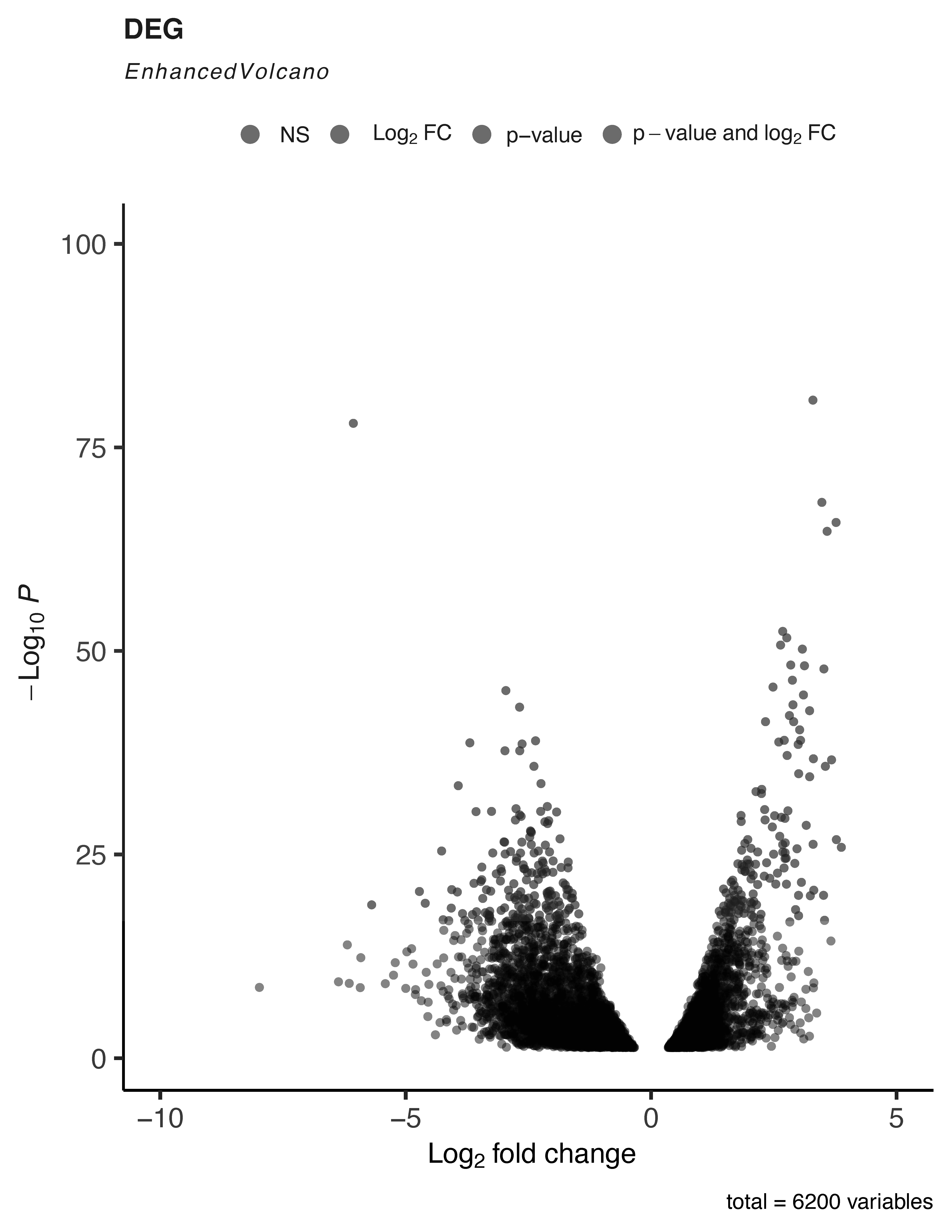


EtOH_TRAP/Control_TRAP (EtOH+ABT-888)_TRAP/Control_TRAP


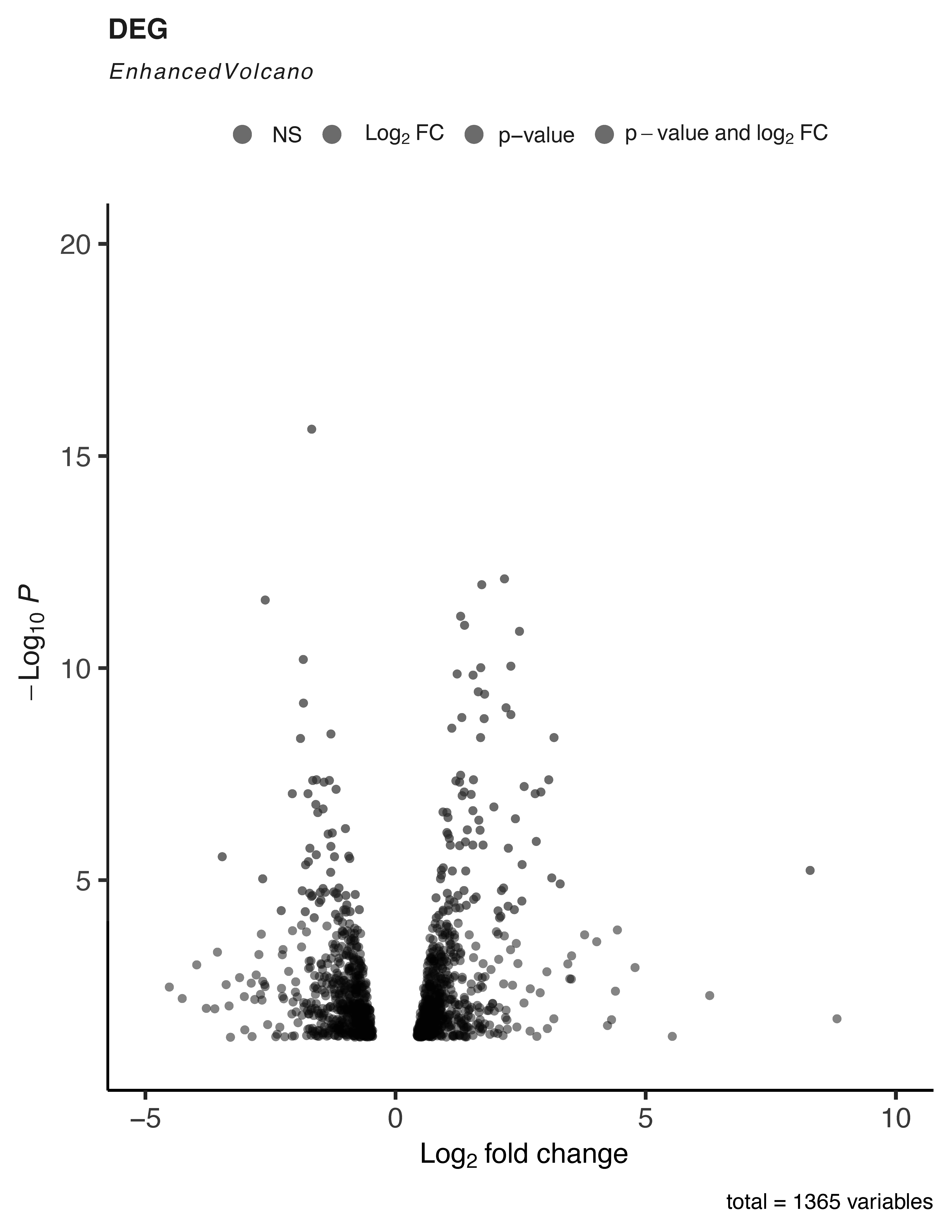

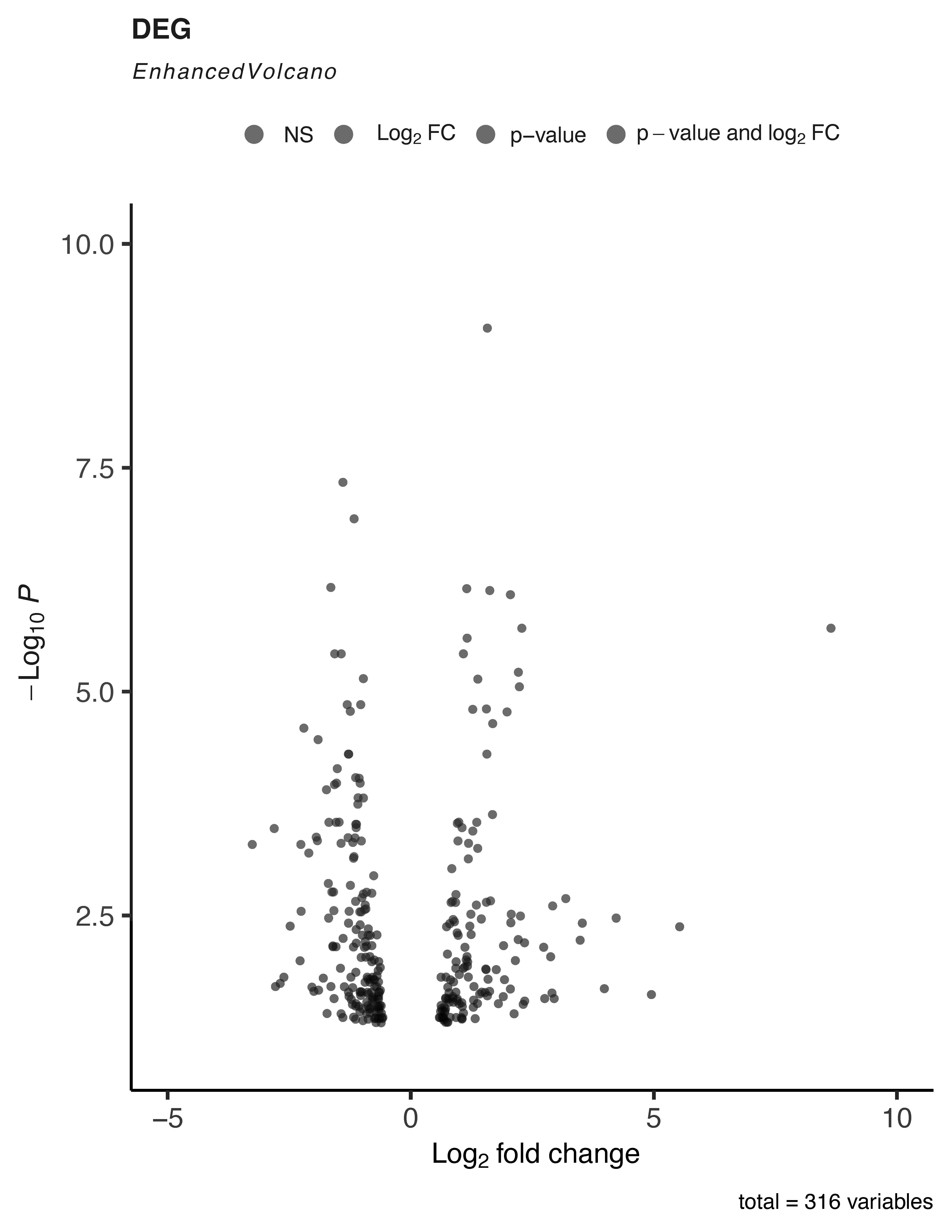


EtOH_INPUT/Control_INPUT (EtOH+ABT-888)_INPUT/Control_INPUT


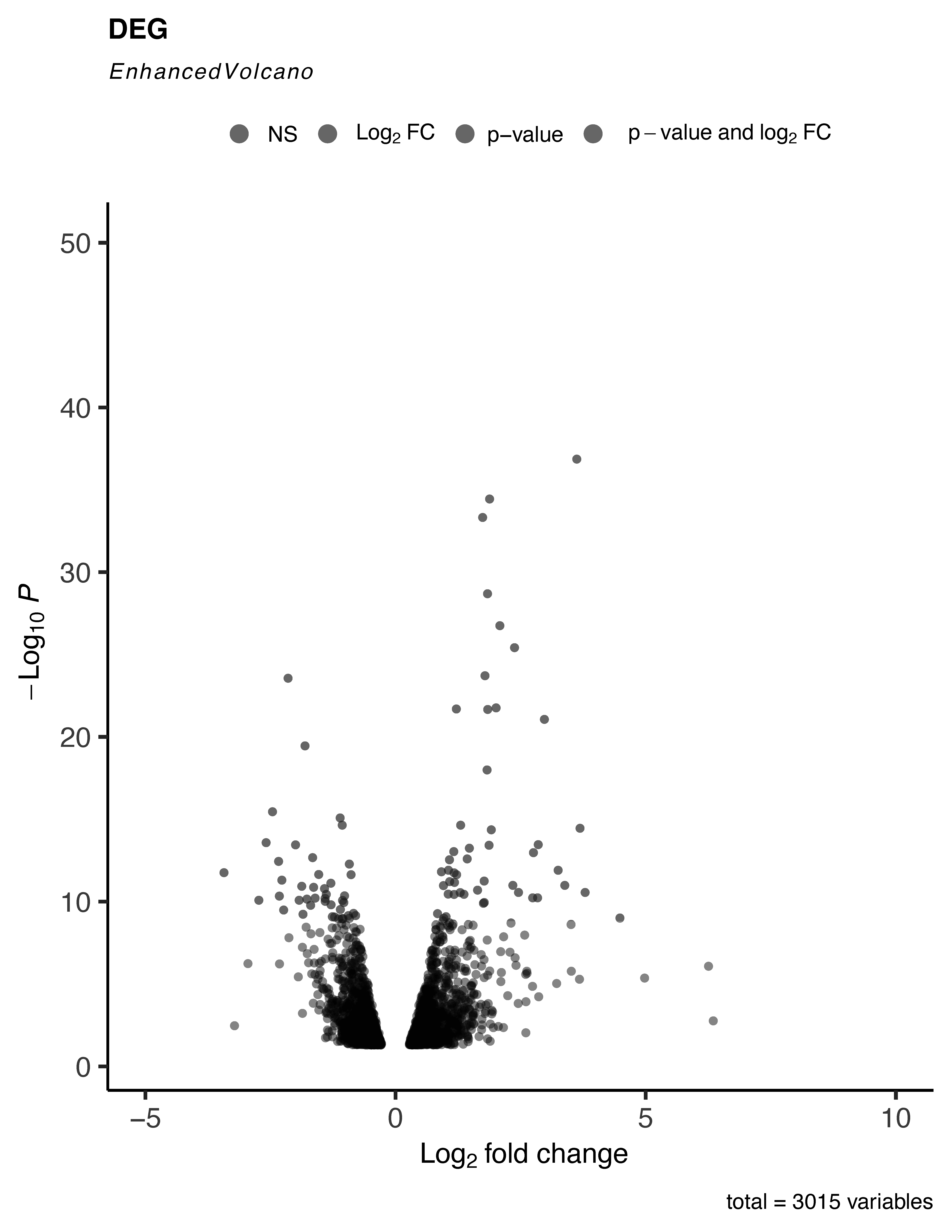

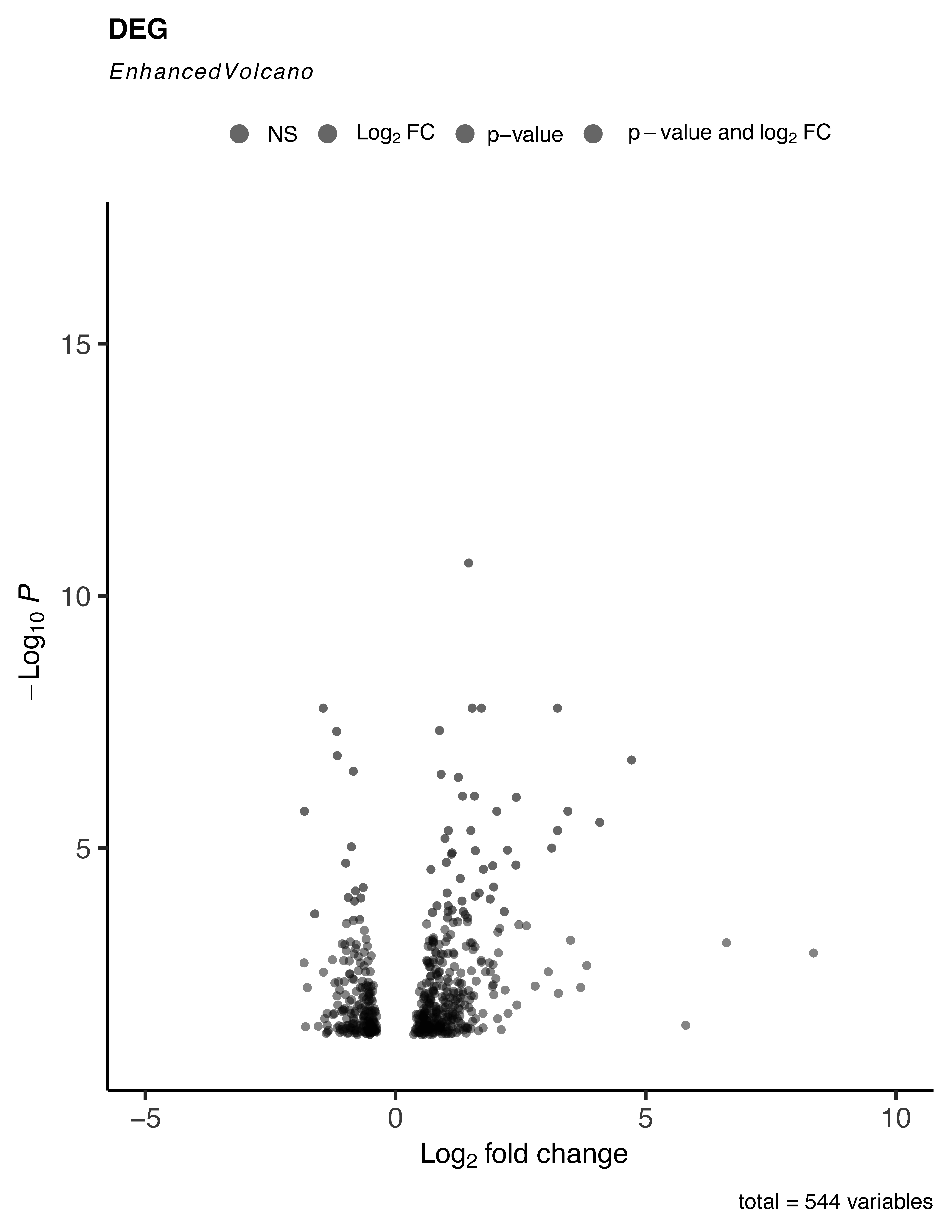


**Supplement Figure 1: Volcano Plots representing differentially expressed transcripts based on seven comparisons from Table 1.**

The log2 fold changes (x) are compared to -log10 (FDR) values (y).


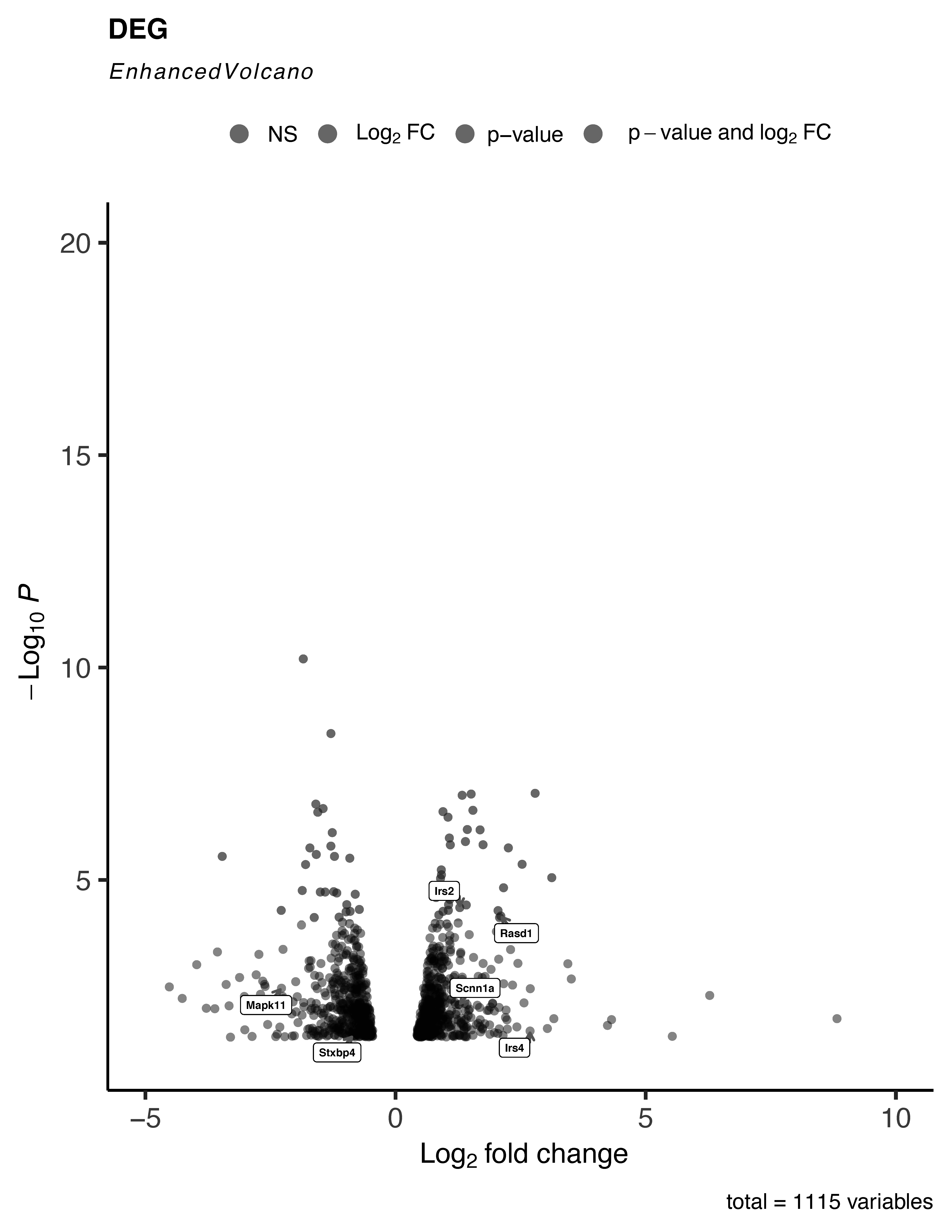


**Supplement Figure 2: Reversal by ABT-888**

The volcano plot shows the 1115 transcripts reversed by ABT-888 administration alongwith the gene names that are part of Insulin Receptor Signaling, whose expression was validated by qRT-PCR.

All volcano plots generated using package EnhancedVolcano in R:

Blighe K, Rana S, Lewis M (2022). EnhancedVolcano: Publication-ready volcano plots with enhanced colouring and labeling. R package version 1.14.0, https://github.com/kevinblighe/EnhancedVolcano.

**Supplement Figure 3: Reversal by ABT-888**

The network images generated using IPA® from the list of genes that are reversed by ABT-888, show two networks that include *Irs4* and *Irs2* and their interacting molecules.

**Supplement Figure 4: Control (Saline), Ethanol and ABT-888 animal treatment protocol**

Male transgenic mice 8-12 weeks old were randomly assigned to three treatment groups: control, ethanol (EtOH), and ethanol + ABT-888 (EtOH+ABT-888). The animals were administered i.p. twice a day with normal saline (CTL) or ethanol (EtOH) for four consecutive days (2h between the first and second injection of the day). ABT-888 (25 mg/kg) was co-administered with ethanol on the fourth day in a sub-group of mice that received ethanol in the previous three days (EtOH+ABT). Two hours after the second injection on day 4, mice were sacrificed via rapid CO2 asphyxiation and decapitation and brains collected.

| Ccr5_F | ggaggtgagacatccgtTc |
| --- | --- |
| Ccr5_R | gagctgagccgcaatttgtt |
| Irs2_F | tgaaggaagccacagtcgtg |
| Irs2_R | tcggaaacatgccaagcatc |
| Irs4_F | ggactttgccagacgagact |
| Irs4_R | tgtgtttggttttggtggcag |
| Mapk11_F | ttctaccggcaagagctgaa |
| Mapk11_R | gggcgagacagcttctttaca |
| Rasd1_F | ggcgaagtctaccagttgga |
| Rasd1_R | ctcttcgaatgagtcgcggt |
| Scnn1a_F | ctaccccgtgagtctcaaca |
| Scnn1a_R | tcaaaaagcgtctgttccgtg |
| Stxbp4_F | acaggaatgaaggaccgctg |
| Stxbp4_R | tctcctggcttcaaacgtcc |

**Supplement Table 1:** **Primers used for mRNA qRT-PCR validations**

**A**

| **Ingenuity Canonical Pathways** | **-log(p-value)** | **Molecules** |
| --- | --- | --- |
| Phagosome Formation | 4.4 | ADGRL4,AGTR1,CCR5,DRD5,FCGR2A,GPR34,GRPR,ITGA1,  ITGA8,LCAT,LPAR1,LPAR6,NMBR,P2RY12,PLD5,SPHK1 |
| Breast Cancer Regulation by Stathmin1 | 2.42 | ADGRL4,AGTR1,CAMK2D,CCR5,DRD5,GPR34,  GRPR,LPAR1,LPAR6,NMBR,P2RY12 |
| CREB Signaling in Neurons | 2.35 | ADGRL4,AGTR1,CAMK2D,CCR5,DRD5,GPR34,  GRPR,LPAR1,LPAR6,NMBR,P2RY12 |
| G-Protein Coupled Receptor Signaling | 2.31 | ADGRL4,AGTR1,CAMK2D,CCR5,DRD5,GPR34,  GRPR,LPAR1,LPAR6,MRTFA,NMBR,P2RY12 |
| Chondroitin Sulfate Biosynthesis | 2.08 | HS3ST3B1,NDST3,XYLT2 |
| Dermatan Sulfate Biosynthesis | 2.04 | HS3ST3B1,NDST3,XYLT2 |
| Apelin Cardiac Fibroblast Signaling Pathway | 1.93 | AGTR1,SPHK1 |
| LXR/RXR Activation | 1.92 | AGT,LCAT,NR1H3,TTR |
| RHOA Signaling | 1.91 | ARHGAP12,LPAR1,LPAR6,RHPN2 |
| Xenobiotic Metabolism PXR Signaling Pathway | 1.89 | CAMK2D,DNAJC7,HS3ST3B1,NDST3,UGT8 |

**B**

| **Ingenuity Canonical Pathways** | **-log(p-value)** | **Molecules** |
| --- | --- | --- |
| Neuropathic Pain Signaling In Dorsal Horn Neurons | 5 | CAMK2D,GRM2,KCNN1,PIK3CD,PLCD3,PLCE1,  PRKCH,TAC1 |
| PI3K Signaling in B Lymphocytes | 3.87 | CAMK2D,IGHM,IL4R,Irs4,JUN,PIK3CD,PLCD3,PLCE1 |
| Uracil Degradation II (Reductive) | 3.5 | DPYD,UPB1 |
| Thymine Degradation | 3.5 | DPYD,UPB1 |
| p70S6K Signaling | 3.29 | AGT,IGHM,IL4R,PIK3CD,PLCD3,PLCE1,PRKCH |
| CREB Signaling in Neurons | 3.23 | ADRA1B,CAMK2D,CHRM3,FLT1,GPR101,GPR149,  GRM2,HTR2C,NPY2R,P2RY13,PIK3CD,PLCD3,PLCE1,  PRKCH,S1PR1,Trhr2 |
| Axonal Guidance Signaling | 3.06 | ADAM32,ADAMTS10,ADAMTS9,ECEL1,EPHB2,EPHB6,  MET,NRP2,PIK3CD,PLCD3,PLCE1,PRKCH,SEMA7A,WNT4 |
| G-Protein Coupled Receptor Signaling | 2.99 | ADRA1B,CAMK2D,CHRM3,ENPP1,GPR101,GPR149,  GRM2,HTR2C,JUN,KCNN1,NPY2R,P2RY13,PIK3CD,  PLCE1,S1PR1,SIX3,Trhr2 |
| Synaptogenesis Signaling Pathway | 2.78 | CAMK2D,CDH1,CDH24,CDH5,EPHB2,EPHB6,GRM2,  PIK3CD,STX1A,TIAM1 |
| Acute Phase Response Signaling | 2.5 | AGT,ITIH3,JUN,PIK3CD,RBP4,Saa3,TTR |

**Supplement Table 2:** **Comparison of EtOH and EtOH+ABT-888**

Using an unadjusted p-value (<0.05) we compared EtOH and EtOH+ABT-888. Shown are the top 10 canonical pathways emerging from the TRAP sample comparisons **(A)** and Input Sample comparisons **(B)**.

**Supplement File 1:** Excel file showing differential expression statistics for all comparisons used in the manuscript.
